# On the Analysis of Wealth Distribution in the Context of Infectious Diseases

**DOI:** 10.3390/e26090788

**Published:** 2024-09-14

**Authors:** Tingting Zhang, Shaoyong Lai, Minfang Zhao

**Affiliations:** 1 School of Mathematics, Southwestern University of Finance and Economics, Chengdu 611130, China; ztt@smail.swufe.edu.cn (T.Z.); laishaoy@swufe.edu.cn (S.L.); 2 School of Mathematics and Statistics, Yili Normal University, Yining 835000, China

**Keywords:** kinetic models, Fokker–Planck equations, wealth distribution, compartmental models in epidemiology, numerical analysis

## Abstract

A mathematical model is established to investigate the economic effects of infectious diseases. The distribution of wealth among two types of agents in the context of the epidemic is discussed. Using the method of statistical mechanics, the evolution of the entropy weak solutions for the model of the susceptible and the infectious involving wealth density functions is analyzed. We assume that as time tends to infinity, the wealth density function of the infectious is linearly related to the wealth density function of the susceptible individuals. Our results indicate that the spreading of disease significantly affects the wealth distribution. When time tends to infinity, the total wealth density function behaves as an inverse gamma distribution. Utilizing numerical experiments, the distribution of wealth under the epidemic phenomenon and the situation of wealth inequality among agents are discussed.

## 1. Introduction

To explore the socio-economic impact of epidemics, Chahuán-Jiménez et al. [1] develop statistical models to study the impact of health on the economy, considering major stock market and health indices. It is found in [1] that the health crisis has varying degrees of impact across different countries. Wang et al. [2] propose a binary wealth interaction rule relating to the psychology of agents to consider the wealth distribution of agents in the context of the epidemic. Goenka et al. [3] establish a theoretical framework to model interaction between income and disease prevalence by combining epidemiological dynamics. Bai et al. [4] set up a mathematical model to investigate impact between COVID-19 and economy, focusing on the interaction between disease transmission and economic growth. Camera and Gioffré [5] construct an analytical framework and illustrate how epidemics affect economic activity. Integrating epidemiological dynamics with kinetic modeling of population-based contacts, Dimarco et al. [6] investigate the evolution of the Boltzmann-type equation over time and describe wealth density functions of the susceptible, infectious, and recovered individuals. Hosseinpoor et al. [7] compare the morbidity and wealth inequality between low-income and middle-income countries. Their results show that wealth inequality is more pronounced in the low-income group than in the middle-income group. Zhang et al. [8] consider the large impact of the rapid diffusion of the coronavirus (COVID-19) on global financial markets.

Many scholars use the theory of rarefied gas dynamics to discuss the distribution of wealth. Bernardi et al. [9] investigate the distribution of agency wealth in the context of infectious diseases, with a particular focus on the economic benefits resulting from vaccination campaigns. Düring and Toscani [10] investigate the saving tendency of different groups in different countries and find that wealth distribution develops a bimodal shape. Quevedo and Quimbay [11] construct a Boltzmann-type model that depicts the variations of wealth distribution. Through a binary trading process, Toscani et al. [12] introduce the dynamical equation associated with the evolution of wealth distribution, illustrating that the solution of the equation is connected to the Pareto tail. Using the classical methods of kinetic theory, Toscani et al. [13] describe the behavior of gamblers in the gambling market. Pareschi and Toscani [14] discuss the large-time behavior of the Boltzmann-type dynamics model. It is proved in [14] that the dynamical model has a nontrivial quasi-steady state with a power-law tail. Della et al. [15] concern a behavioral epidemiological model, which is relevant to the kinetic theory. Loy and Tosin [16] investigate a viral load-based model and discuss the spread of the epidemic.

In the context of epidemiology, this paper examines a model of wealth exchange. Hethcote [17] analyzes the forms of transmission of infectious diseases among agents. Hethcote [17] extends the classical Susceptibles-Infectives-Recovered (SIR) and Susceptibles-infection-Susceptibles (SIS) epidemiological model. The SIR epidemiological model divides the agents into three categories: susceptible (*S*), infectious (*I*), and recovered (*R*). The SIS epidemiological model divides the agents into two categories: susceptible (*S*) and infectious (*I*). Cordier et al. [18] discuss a nonlinear dynamic model of a market economy based on binary trading. It is proved in [18] that as time tends to infinity, the nonlinear dynamical model is well approximated to the linear Fokker–Planck equation. To explore the socio-economic impact of epidemics, Dimarco et al. [19] combine the SIR model in [17] with the wealth exchange model in [18], which assumes that the agents gain some immunity after recovery, meaning that they do not become susceptible again. However, there are some diseases that cause the agents not to have immunity after recovery.

We introduce that the probability density functions of the susceptible and the infectious are $H_{S}\left(t,w\right)$ and $H_{I}\left(t,w\right)$, respectively. *w* represents the agent’s wealth, w>0, and *t* represents time. We assume that the evolution of the densities obeys the classical SIS model in [17]. From Hethcote [17], we know that when time tends to infinity, the number of the infectious I(t) is related to the ratio of β and γ (β is the interaction rate of infections and γ is the recovery rate of agents, β and γ are constants, 0<β<1, 0<γ<1). In this work, due to the non-negativity of the density function, when β≤γ, we have $\lim_{t\to \infty }H_{I}^{\infty }\left(w\right)=0$. When β>γ, we have $\lim_{t\to \infty }H_{I}^{\infty }\left(w\right)\neq 0$. Namely, we discuss the situation of $\lim_{t\to \infty }H_{I}^{\infty }\left(w\right)\neq 0$. $H_{I}^{\infty }\left(w\right)$ means $H_{I}\left(t,w\right)$ when t→∞. On the basis of the works in Refs. [17,18], our contributions are as follows.

(i) In this paper, we combine the SIS model in [17] with the wealth exchange model in [18], which assumes that the agents have no immunity. This means that agents become susceptible again after recovery.
(ii) Our trading rules are different from those in [18]. The trading rule in [18] introduces a random variable η, requiring its mathematical expectations $E\left(\eta_{1}\right)=E\left(\eta_{2}\right)=0$. In this work, we consider the effect of differences of wealth between agents during infectious diseases by setting $E\left(\eta_{1}\right)=\zeta \frac{w_{*}-w}{w}$ and $E\left(\eta_{2}\right)=\zeta \frac{w-w_{*}}{w_{*}}$, where ζ is a proportional constant $w_{*}$ and *w* stand for the wealth of two agents (for a detailed explanation of $E\left(\eta_{1}\right)=\zeta \frac{w_{*}-w}{w}$ and $E\left(\eta_{2}\right)=\zeta \frac{w-w_{*}}{w_{*}}$, see Boghosian et al. [20]).
(iii) We obtain steady-state solutions that differ from those in [18]. Our steady-state solution is related to the proportionality constant ζ, which is not discussed in [18].

The structure of this article is as follows: In Section 2, we introduce the wealth model in the epidemiological model, giving the rules of transactions between agents. In Section 3 and Section 4, we provide a steady-state solution for the susceptible and the infectious. In Section 5, numerical analysis of the steady-state solutions is provided.

## 2. Wealth Dynamics in Epidemiologic Models

In this section, we introduce a dynamic system of agents’ wealth within the framework of the infectious disease. We assume that agents are indistinguishable [21]. In the classical SIS model [17], the agents are divided into two categories: the susceptible, who can contract the disease; the infectious, who have contracted disease and can transmit diseases. The SIS model assumes that agents are not immune after recovery and can be infected again. This means that the agents become susceptible again after recovery. The probability density function of these two types of agents is $H_{S}\left(t,w\right)$ and $H_{I}\left(t,w\right)$ (*w* stands for wealth and $w\in R_{+}$), respectively. The total wealth density is

$$H\left(t,w\right)=H_{S}\left(t,w\right)+H_{I}\left(t,w\right).$$

In general, we assume that the density function satisfies $\int_{R_{+}}H\left(t,w\right)dw=1.$ We define

$$S\left(t\right)=\int_{R^{+}}H_{S}\left(t,w\right)dw,\;I\left(t\right)=\int_{R^{+}}H_{I}\left(t,w\right)dw,$$

where S(t) and I(t) denote the proportion of agents who are susceptible and infectious, respectively. We know

(1) S(t)+I(t)=1.

We write out the total average wealth as

$$m\left(t\right)=\int_{R^{+}}wH\left(t,w\right)dw$$

and corresponding wealths for the two types of agents are

$$\begin{array}{cc} \; & m_{S}\left(t\right)=\int_{R^{+}}wH_{S}\left(t,w\right)dw,\\ & m_{I}\left(t\right)=\int_{R^{+}}wH_{I}\left(t,w\right)dw. \end{array}$$

Thus, we have

(2) $$m_{S}\left(t\right)+m_{I}\left(t\right)=m\left(t\right).$$

Suppose that an agent intends to make an investment or trade. Typically, this type of investment carries a certain amount of risk, either providing the buyer with additional wealth or leading to a loss of wealth in an uncertain manner. According to the pre-trade rules in [18], we assume that the change in the agent’s wealth before and after the transaction is

(3) $$\begin{array}{c} \left\{\begin{array}{c} w^{\prime }=w+\lambda \left(w_{*}-w\right)+\eta_{1}w,\\ w_{*}^{\prime }=w_{*}+\lambda \left(w-w_{*}\right)+\eta_{2}w_{*}, \end{array}\right. \end{array}$$

in which $w^{\prime }$ and $w_{*}^{\prime }$ denote the wealth after the transaction, *w* and $w_{*}$ are the wealth before the transaction. $\eta_{1}$ and $\eta_{2}$ are independent random variables. λ (0<λ<1) represents the transaction coefficient, implying the intuitive behavior that prevents the agent from investing the entire amount in a single transaction (more details can be found in [2,6,19,22]).

Based on the idea of Boghosian et al. [20], we assume. (In the following discussion of this paper, we use $\langle \cdot \rangle$ to denote the mathematical expectation).

$$\begin{array}{cc} \; & \langle \eta_{1}\rangle =\zeta \frac{w_{*}-w}{w},\;\langle \eta_{2}\rangle =\zeta \frac{w-w_{*}}{w_{*}},\;\langle \eta_{1}^{2}\rangle =\langle \eta_{2}^{2}\rangle =\sigma ,\\ \; & \left|\langle \eta_{1}^{3}\rangle \right|\leq C_{0}\left(\zeta^{2}+\sigma^{2}\right),\;\left|\langle \eta_{2}^{3}\rangle \right|\leq C_{0}\left(\zeta^{2}+\sigma^{2}\right), \end{array}$$

where $\frac{w_{*}-w}{w}$ and $\frac{w-w_{*}}{w_{*}}$ explain the wealth difference between agents. $C_{0}>0$, and σ>0 are constants. We take $\frac{w_{*}-w}{w}$ as an example. If the ratio of $\frac{w_{*}-w}{w}$ is greater than 1, meaning that wealth $w_{*}$ is larger than wealth *w*, indicating a large wealth difference between agents. If $\frac{w_{*}-w}{w}$ is closer to 0, implying that the wealth difference between agents is smaller. $\langle \eta_{1}\rangle$ and $\langle \eta_{2}\rangle$ are related to a proportional constant variable ζ, 0<ζ<1.

From Equation (3), we have

(4) $$\begin{array}{c} \langle w^{\prime }+w_{*}^{\prime }\rangle =w+w_{*}, \end{array}$$

meaning that total wealth is conserved.

We assume that density evolution follows the classical SIS model [17]

(5) $$\begin{array}{cc} \frac{\partial H_{S}\left(t,w\right)}{\partial t} & =-\Pi \left(t,w\right)H_{S}\left(t,w\right)+\gamma \left(w\right)H_{I}\left(t,w\right)\\ \; & +\sum_{J\in \{S,I\}}K\left(H_{S},H_{J}\right)\left(t,w\right),\;J\in \left\{S,I\right\}, \end{array}$$

(6) $$\begin{array}{cc} \frac{\partial H_{I}\left(t,w\right)}{\partial t} & =\Pi \left(t,w\right)H_{S}\left(t,w\right)-\gamma \left(w\right)H_{I}\left(t,w\right)\\ \; & +\sum_{J\in \{S,I\}}K\left(H_{I},H_{J}\right)\left(t,w\right),\;J\in \left\{S,I\right\}, \end{array}$$

where

$$\Pi \left(t,w\right)=\int_{R_{+}}\beta \left(w,w_{*}\right)H_{I}\left(t,w_{*}\right)dw_{*},$$

in which the interaction rate of infections $\beta \left(w,w_{*}\right)=\beta$ ( 0<β<1 is a constant). We let the recovery rate of agents γ(w)=γ (0<γ<1 is a constant). Operator *K* (· , ·) describes the evolution of wealth because of the transactions among agents in one class or different classes. In Equations (5) and (6), the evolution of wealth among different classes of agents is described. When a susceptible individual contacts with an infected individual, the former may be transferred to the latter. Since the individual possesses no immunity, it may become the infected agent after recovery. Obviously, the transfer of the agent implies that the wealth owned by the agent is transferred. We are now ready to define the operator *K* (· , ·) in all cases. A convenient way to express an operator is based on its weak form. Let ϕ(w) denote a smooth function with a supported set in $R_{+}$. The wealth density function $H_{I}$ and $H_{S}$ obey the integral equations. Based on the statements in Cordier et al. [18], we have the following equations

(7) $$\begin{array}{cc} \; & \int_{R_{+}}K\left(H_{S},H_{J}\right)\phi \left(w\right)dw\\ \; & =\langle \int_{R_{+}^{2}}H_{S}\left(t,w\right)H_{J}\left(t,w_{*}\right)\left(\phi \left(w^{\prime }\right)-\phi \left(w\right)\right)dwdw_{*}\rangle , \end{array}$$

(8) $$\begin{array}{c} \int_{R_{+}}K\left(H_{I},H_{J}\right)\phi \left(w\right)dw\\ =\langle \int_{R_{+}^{2}}H_{I}\left(t,w\right)H_{J}\left(t,w_{*}\right)\left(\phi \left(w^{\prime }\right)-\phi \left(w\right)\right)dwdw_{*}\rangle , \end{array}$$

where $w^{\prime }$ is defined as in (3) and $\langle \cdot \rangle$ stands for mathematical expectation. Thus, Equations (5) and (6) become

(9) $$\begin{array}{cc} \; & \frac{d}{dt}\int_{R_{+}}H_{S}\left(t,w\right)\phi \left(w\right)dw\\ \; & =-\beta I\left(t\right)\int_{R_{+}}H_{S}\left(t,w\right)\phi \left(w\right)dw\\ \; & +\gamma \int_{R_{+}}H_{I}\left(t,w\right)\phi \left(w\right)dw\\ \; & +\sum_{J\in \{S,I\}}\int_{R_{+}}K\left(H_{S},H_{J}\right)\left(t,w\right)\phi \left(w\right)dw, \end{array}$$

(10) $$\begin{array}{cc} \; & \frac{d}{dt}\int_{R_{+}}H_{I}\left(t,w\right)\phi \left(w\right)dw\\ \; & =\beta I\left(t\right)\int_{R_{+}}H_{S}\left(t,w\right)\phi \left(w\right)dw\\ \; & -\gamma \int_{R_{+}}H_{I}\left(t,w\right)\phi \left(w\right)dw\\ \; & +\sum_{J\in \{S,I\}}\int_{R_{+}}K\left(H_{I},H_{J}\right)\left(t,w\right)\phi \left(w\right)dw. \end{array}$$

Using Equations (9) and (10) yields

$$\begin{array}{cc} \; & \frac{d}{dt}\int_{R_{+}}H\left(t,w\right)\phi \left(w\right)dw\\ \; & =\frac{d}{dt}\int_{R_{+}}H_{S}\left(t,w\right)\phi \left(w\right)dw+\frac{d}{dt}\int_{R_{+}}H_{I}\left(t,w\right)\phi \left(w\right)dw\\ \; & =\sum_{J\in \{S,I\}}\int_{R_{+}}K\left(H_{S},H_{J}\right)\left(t,w\right)\phi \left(w\right)dw\\ \; & +\sum_{J\in \{S,I\}}\int_{R_{+}}K\left(H_{I},H_{J}\right)\left(t,w\right)\phi \left(w\right)dw. \end{array}$$

When ϕ(w)=1, we obtain

$$\frac{d}{dt}\int_{R_{+}}H\left(t,w\right)\phi \left(w\right)dw=0.$$

We obtain the conservation nature. This means that the number of general agents remains the same over time.

When ϕ(w)=w and using Equation (4), we have

$$\begin{array}{cc} \; & \int_{R_{+}}K\left(H_{S},H_{J}\right)\phi \left(w\right)dw\\ \; & =\langle \int_{R_{+}^{2}}\left(\phi \left(w^{\prime }\right)-\phi \left(w\right)\right)H_{S}\left(t,w\right)H_{J}\left(t,w_{*}\right)dwdw_{*}\rangle \\ \; & =\frac{1}{2}\langle \int_{R_{+}^{2}}\left(w^{\prime }+w_{*}^{\prime }\right)H_{S}\left(t,w\right)H_{J}\left(t,w_{*}\right)dwdw_{*}\rangle \\ \; & -\frac{1}{2}\langle \int_{R_{+}^{2}}\left(w+w_{*}\right)H_{S}\left(t,w\right)H_{J}\left(t,w_{*}\right)dwdw_{*}\rangle \\ \; & =0. \end{array}$$

Thus, we acquire

$$\int_{R_{+}}K\left(H_{S},H_{J}\right)\phi \left(w\right)dw=\int_{R_{+}}K\left(H_{I},H_{J}\right)\phi \left(w\right)dw=0.$$

and

$$\frac{d}{dt}\int_{R_{+}}H\left(t,w\right)wdw=0.$$

This means that average wealth is conserved.

Substituting ϕ(w)=1 into Equations (9) and (10), we obtain that the fractions of the susceptible and infectious satisfy

(11) $$\begin{array}{c} \left\{\begin{array}{c} \frac{dS(t)}{dt}=-\beta S\left(t\right)I\left(t\right)+\gamma I\left(t\right),\\ \frac{dI(t)}{dt}=\beta S\left(t\right)I\left(t\right)-\gamma I\left(t\right). \end{array}\right. \end{array}$$

System (11) is a classical SIS model in [17].

**Theorem 1 **([17]).****  *Let (S(t),I(t)) be a solution of the classical SIS model in*

{(S(t),I(t))∣S(t)≥0,I(t)≥0,S(t)+I(t)=1}.

*If $\frac{\beta }{\gamma }\leq 1$, then I(t) decreases to zero as t→∞. If $\frac{\beta }{\gamma }>1$, then I(t) approaches $1-\frac{\beta }{\gamma }$ as t→∞.*

Theorem 1 implies that when $\frac{\beta }{\gamma }\leq 1$, the number of the infectious I(t) at steady state (i.e., as t→∞) tends to zero. Combined with the non-negativity of the distribution function, the wealth distribution of the group approaches zero, i.e., $H_{I}^{\infty }=0$. When $\frac{\beta }{\gamma }>1$, the number of the infectious I(t) tends to a stable value (i.e., as t→∞). In this case, $H_{I}^{\infty }$ is not equal to 0.

## 3. When $\frac{\beta }{\gamma }>1$

### 3.1. Steady-State Solution of $H_{S}\left(w,t\right)$

If we want to ask for a solution about $H_{S}\left(w,t\right)$, we need to start with Equation (5). From Equation (5), we obtain

$$\begin{array}{cc} \; & \int_{R^{+}}\frac{\partial H_{S}\left(t,w\right)}{\partial t}\phi \left(w\right)dw\\ \; & =-\beta I\left(t\right)\int_{R_{+}}H_{S}\left(t,w\right)\phi \left(w\right)dw\\ \; & +\gamma \int_{R_{+}}H_{I}\left(t,w\right)\phi \left(w\right)dw\\ \; & +\int_{R_{+}}\sum_{J\in \{S,I\}}K\left(H_{S},H_{J}\right)\left(t,w\right)\phi \left(w\right)dw. \end{array}$$

Following the ideas in [18], we scale the binary trades by setting

$$\begin{array}{c} \lambda \to \varepsilon \lambda ,\;\sigma \to \varepsilon \sigma ,\;\zeta \to \varepsilon \zeta . \end{array}$$

Using interaction rule (3), expanding Taylor series $\phi (w^{\prime })$ around ϕ(w), and noticing the properties of stochastic variables $\eta_{1}$ and $\eta_{2}$, we obtain

$$\begin{array}{cc} \; & \langle w^{\prime }-w\rangle =\langle \lambda \left(w_{*}-w\right)+\eta_{1}w\rangle =\varepsilon \left(\zeta +\lambda \right)\left(w_{*}-w\right),\\ \; & \langle {\left(w^{\prime }-w\right)}^{2}\rangle =\varepsilon^{2}\left(\lambda^{2}+2\lambda \zeta \right){\left(w_{*}-w\right)}^{2}+\varepsilon \sigma w^{2} \end{array}$$

and

$$\begin{array}{cc} \; & \langle \phi \left(w^{\prime }\right)-\phi \left(w\right)\rangle \\ \; & =\phi^{\prime }\left(w\right)\langle w^{\prime }-w\rangle +\frac{\phi^{\prime \prime }\left(w\right)}{2}\langle {\left(w^{\prime }-w\right)}^{2}\rangle +r_{\varepsilon }\left(w\right)\\ \; & =\varepsilon \left(\phi^{\prime }\left(w\right)\left(\zeta +\lambda \right)\left(w_{*}-w\right)+\phi^{\prime \prime }\left(w\right)\frac{\sigma }{2}w^{2}\right)+r_{\varepsilon }\left(w\right), \end{array}$$

in which the term $r_{\varepsilon }\left(w\right)$ satisfies

$$\begin{array}{cc} \; & r_{\varepsilon }\left(w\right)=\varepsilon^{2}\left(\lambda^{2}+2\lambda \zeta \right){\left(w_{*}-w\right)}^{2}\\ \; & +\frac{1}{6}\langle \phi^{\prime \prime \prime }\left(w+\theta \left(w^{\prime }-w\right)\right){\left(w^{\prime }-w\right)}^{3}\rangle , \end{array}$$

where 0≤θ≤1.

In fact, for a small ε, readjusting the time to $t\to \frac{t}{\varepsilon }$ and avoiding the dependence and variance of time on the mean, from Equation (7), we obtain

$$\begin{array}{cc} \; & \frac{1}{\varepsilon }\int_{R_{+}}K_{\varepsilon }\left(H_{S},H_{J}\right)\phi \left(w\right)dw\\ \; & =\int_{R_{+}^{2}}H_{S}\left(t,w\right)H_{J}\left(t,w_{*}\right)\phi^{\prime }\left(w\right)\left(\zeta +\lambda \right)\left(w_{*}-w\right)dwdw_{*}\\ \; & +\int_{R_{+}^{2}}H_{S}\left(t,w\right)H_{J}\left(t,w_{*}\right)\phi^{\prime \prime }\left(w\right)\frac{\sigma }{2}w^{2}dwdw_{*}+\frac{R_{\varepsilon }\left(t\right)}{\varepsilon }, \end{array}$$

where

$$\begin{array}{c} R_{\varepsilon }\left(t\right)=\int_{R_{+}^{2}}r_{\varepsilon }\left(w\right)H_{\varepsilon S}\left(t,w\right)H_{\varepsilon J}\left(t,w_{*}\right)dwdw_{*}. \end{array}$$

It is easy to derive the remainder term $R_{\varepsilon }\left(t\right)$ satisfies $\frac{1}{\varepsilon }R_{\varepsilon }\left(t\right)\to 0$ as ε→0. Therefore, we obtain the equation

$$\begin{array}{cc} \; & \frac{1}{\varepsilon }\int_{R_{+}}K_{\varepsilon }\left(H_{S},H_{J}\right)\phi \left(w\right)dw\\ \; & =\int_{R_{+}^{2}}H_{S}\left(t,w\right)H_{J}\left(t,w_{*}\right)\phi^{\prime }\left(w\right)\left(\zeta +\lambda \right)\left(w_{*}-w\right)dwdw_{*}\\ & +\int_{R_{+}^{2}}H_{S}\left(t,w\right)H_{J}\left(t,w_{*}\right)\phi^{\prime \prime }\left(w\right)\frac{\sigma }{2}w^{2}dwdw_{*}, \end{array}$$

in which

$$\begin{array}{cc} \; & \int_{R_{+}^{2}}H_{S}\left(t,w\right)H_{J}\left(t,w_{*}\right)\phi^{\prime }\left(w\right)\left(\zeta +\lambda \right)\left(w_{*}-w\right)dwdw_{*}\\ \; & =\left(\zeta +\lambda \right)\int_{R_{+}^{2}}H_{S}\left(t,w\right)H_{J}\left(t,w_{*}\right)\left(w_{*}-w\right)\phi^{\prime }\left(w\right)dwdw_{*}\\ \; & =\left(\zeta +\lambda \right)\int_{R_{+}^{2}}H_{S}\left(t,w\right)H_{J}\left(t,w_{*}\right)w_{*}\phi^{\prime }\left(w\right)dwdw_{*}\\ \; & -\left(\zeta +\lambda \right)\int_{R_{+}^{2}}H_{S}\left(t,w\right)H_{J}\left(t,w_{*}\right)w\phi^{\prime }\left(w\right)dwdw_{*}. \end{array}$$

We write

$$\begin{array}{cc} m_{J}\left(t\right) & =\int_{R_{t}}\omega_{*}H_{J}\left(t,w_{*}\right)dw_{*}=\int_{R_{t}}wH_{J}\left(t,w\right)d\omega ,\;J=\left\{S,I\right\},\\ J(t) & =\int_{R_{t}}H_{J}\left(t,w_{*}\right)dw_{*}=\int_{R_{t}}H_{J}\left(t,w\right)dw,\;J=\left\{S,I\right\}. \end{array}$$

Using the properties of ϕ(w) and integration by parts, we derive that

$$\begin{array}{cc} \; & \int_{R_{+}^{2}}H_{S}\left(t,w\right)H_{J}\left(t,w_{*}\right)\phi^{\prime }\left(w\right)\left(\zeta +\lambda \right)\left(w_{*}-w\right)dwdw_{*}\\ \; & =m_{J}\left(t\right)\left(\zeta +\lambda \right)\int_{R_{+}}H_{S}\left(t,w\right)\phi^{\prime }\left(w\right)dw\\ \; & -J\left(t\right)\left(\zeta +\lambda \right)\int_{R_{+}}wH_{S}\left(t,w\right)\phi^{\prime }\left(w\right)dw\\ \; & =m_{J}\left(t\right)\left(\zeta +\lambda \right)\int_{R_{+}}H_{S}\left(t,w\right)d\phi \left(w\right)\\ & -\frac{J(t)(1-\lambda )}{2}\int_{R_{+}}wH_{S}\left(t,w\right)d\phi \left(w\right)\\ \; & =-m_{J}\left(t\right)\left(\zeta +\lambda \right)\int_{R_{+}}\frac{\partial \left(H_{S}\left(t,w\right)\right)}{\partial w}\phi \left(w\right)dw\\ \; & +J\left(t\right)\left(\zeta +\lambda \right)\int_{R_{+}}\frac{\partial \left(wH_{S}\left(t,w\right)\right)}{\partial w}\phi \left(w\right)dw \end{array}$$

and

$$\begin{array}{cc} \; & \int_{R_{+}^{2}}H_{S}\left(t,w\right)H_{J}\left(t,w_{*}\right)\frac{\phi^{\prime \prime }\left(w\right)}{2}\sigma w^{2}dwdw_{*}\\ \; & =\frac{\sigma J(t)}{2}\int_{R_{+}}w^{2}H_{S}\left(t,w\right)\phi^{\prime \prime }\left(w\right)dw\\ \; & =\frac{\sigma J(t)}{2}\int_{R_{+}}w^{2}H_{S}\left(t,w\right)d\phi^{\prime }\left(w\right)\\ \; & =-\frac{\sigma J(t)}{2}\int_{R_{+}}\frac{\partial \left(w^{2}H_{S}\left(t,w\right)\right)}{\partial w}\phi^{\prime }\left(w\right)dw\\ \; & =-\frac{\sigma J(t)}{2}\int_{R_{+}}\frac{\partial \left(w^{2}H_{S}\left(t,w\right)\right)}{\partial w}d\phi \left(w\right)\\ \; & =\frac{\sigma J(t)}{2}\int_{R_{+}}\frac{\partial^{2}\left(w^{2}H_{S}\left(t,w\right)\right)}{\partial w^{2}}\phi \left(w\right)dw. \end{array}$$

Thus, we have

$$\begin{array}{cc} \; & \int_{R_{+}}K_{\varepsilon }\left(H_{S},H_{J}\right)\phi \left(w\right)dw\\ \; & =\int_{R_{+}}-m_{J}\left(t\right)\left(\zeta +\lambda \right)\int_{R_{+}}\frac{\partial \left(H_{S}\left(t,w\right)\right)}{\partial w}\phi \left(w\right)dw\\ \; & +J\left(t\right)\left(\zeta +\lambda \right)\int_{R_{+}}\frac{\partial \left(wH_{S}\left(t,w\right)\right)}{\partial w}\phi \left(w\right)dw\\ \; & +\int_{R_{+}}\frac{\sigma J(t)}{2}\frac{\partial^{2}}{\partial^{2}w}\left(w^{2}H_{S}\left(t,w\right)\right)\phi \left(w\right)dw. \end{array}$$

Using Equations (1) and (2) yields

$$\begin{array}{cc} \; & \int_{R_{+}}\sum_{J\in \{S,I\}}K\left(H_{S},H_{J}\right)\left(t,w\right)\phi \left(w\right)dw\\ \; & =\int_{R_{+}}-\left(m_{S}\left(t\right)+m_{I}\left(t\right)\right)\left(\zeta +\lambda \right)\frac{\partial \left(H_{S}\left(t,w\right)\right)}{\partial w}\phi \left(w\right)dw\\ \; & +\left(S\left(t\right)+I\left(t\right)\right)\left(\zeta +\lambda \right)\int_{R_{+}}\frac{\partial \left(wH_{S}\left(t,w\right)\right)}{\partial w}\phi \left(w\right)dw\\ \; & +\int_{R_{+}}\frac{\sigma (S(t)+I(t))}{2}\frac{\partial^{2}}{\partial^{2}w}\left(w^{2}H_{S}\left(t,w\right)\right)\phi \left(w\right)dw\\ \; & =\int_{R_{+}}-m\left(t\right)\left(\zeta +\lambda \right)\frac{\partial \left(H_{S}\left(t,w\right)\right)}{\partial w}\phi \left(w\right)dw\\ \; & +\left(\zeta +\lambda \right)\int_{R_{+}}\frac{\partial \left(wH_{S}\left(t,w\right)\right)}{\partial w}\phi \left(w\right)dw\\ \; & +\int_{R_{+}}\frac{\sigma }{2}\frac{\partial^{2}}{\partial^{2}w}\left(w^{2}H_{S}\left(t,w\right)\right)\phi \left(w\right)dw. \end{array}$$

Thus, we obtain

$$\begin{array}{cc} \; & \int_{R^{+}}\frac{\partial H_{S}\left(t,w\right)}{\partial t}\phi \left(w\right)dw\\ \; & =-\beta I\left(t\right)\int_{R_{+}}H_{S}\left(t,w\right)\phi \left(w\right)dw\\ \; & +\gamma \int_{R_{+}}H_{I}\left(t,w\right)\phi \left(w\right)dw\\ \; & +\int_{R_{+}}-m\left(t\right)\left(\zeta +\lambda \right)\frac{\partial \left(H_{S}\left(t,w\right)\right)}{\partial w}\phi \left(w\right)dw\\ \; & +\left(\zeta +\lambda \right)\int_{R_{+}}\frac{\partial \left(wH_{S}\left(t,w\right)\right)}{\partial w}\phi \left(w\right)dw\\ \; & +\int_{R_{+}}\frac{\sigma }{2}\frac{\partial^{2}}{\partial^{2}w}\left(w^{2}H_{S}\left(t,w\right)\right)\phi \left(w\right)dw. \end{array}$$

Then, we acquire

$$\begin{array}{cc} \frac{\partial H_{S}\left(t,w\right)}{\partial t} & =-\beta I\left(t\right)H_{S}\left(t,w\right)+\gamma H_{I}\left(t,w\right)\\ \; & +\frac{\partial }{\partial w}\left[\left(w(\zeta +\lambda )-m(t)(\zeta +\lambda )\right)H_{S}\left(t,w\right)\right]\\ \; & +\frac{\sigma }{2}\frac{\partial^{2}}{\partial^{2}w}\left(w^{2}H_{S}\left(t,w\right)\right). \end{array}$$

When t→∞, we assume that m(t) is a constant *m* and I(t) is a constant $I_{\infty }$, from which we obtain the equation (see Cordier et al. [18])

(12) $$\begin{array}{cc} \; & -\beta I_{\infty }H_{S}^{\infty }+\gamma H_{I}^{\infty }+\left(w(\zeta +\lambda )-(\zeta +\lambda )m\right)H_{S}^{\infty }\\ \; & +\frac{\sigma }{2}\frac{\partial }{\partial w}\left(w^{2}H_{S}^{\infty }\right)=0. \end{array}$$

When t→∞, we assume that

(13) $$\begin{array}{c} H_{I}^{\infty }=DH_{S}^{\infty }, \end{array}$$

where D>0 is a constant. In the SIS model, we assume that agents do not have immunity after infection. Agents switch back and forth between the infectious and susceptible. In this case, the ratio of the infectious to susceptibles tends to a stable state as time approaches infinity. Thus, assumption (13) is reasonable.

From (12) and (13), we obtain

$$\begin{array}{cc} \; & -\beta I_{\infty }H_{S}^{\infty }+\gamma DH_{S}^{\infty }+\left(w(\zeta +\lambda )-(\zeta +\lambda )m\right)H_{S}^{\infty }\\ \; & +\frac{\sigma }{2}\frac{\partial }{\partial w}\left(w^{2}H_{S}^{\infty }\right)=0, \end{array}$$

which is equivalent to

$$\begin{array}{cc} \; & \left[w\left(\zeta +\lambda +\sigma \right)-\left(\zeta +\lambda \right)m-\beta I_{\infty }+\gamma D\right]H_{S}^{\infty }\\ \; & +\frac{\sigma }{2}w^{2}\frac{\partial }{\partial w}\left(H_{S}^{\infty }\right)=0, \end{array}$$

from which we have

$$\begin{array}{c} \left(Aw-B\right)H_{S}^{\infty }+Fw^{2}\frac{\partial }{\partial w}\left(H_{S}^{\infty }\right)=0, \end{array}$$

where

$$\begin{array}{cc} \; & A=\zeta +\lambda +\sigma ,\\ \; & B=\left(\zeta +\lambda \right)m+\beta I_{\infty }-\gamma D,\\ \; & F=\frac{\sigma }{2}. \end{array}$$

Thus, we have the solution

$$\begin{array}{c} H_{S}^{\infty }=e^{-\int \frac{Aw-B}{Fw^{2}}dw}=e^{-\frac{A}{F}\ln w-\frac{B}{Fw}}\cdot C, \end{array}$$

where *C* is a constant. Record the formula $S\left(t\right)=\int_{R^{+}}g_{S}\left(t,w\right)dw$ in the first section. When t→∞, we have

$$\begin{array}{c} S\left(t\right)\to S_{\infty },\;S_{\infty }=\int_{R^{+}}H_{S}^{\infty }dw. \end{array}$$

We obtain

$$\begin{array}{c} C\cdot \int_{0}^{\infty }e^{-\frac{A}{F}\ln w-\frac{B}{Fw}}dw=S_{\infty }. \end{array}$$

Letting $y=\frac{B}{wF}$, we obtain

$$\begin{array}{c} C\cdot {\left(\frac{B}{F}\right)}^{-\frac{A}{F}+1}\Gamma \left(\frac{A}{F}-1\right)=S_{\infty }, \end{array}$$

where $\Gamma \left(\frac{A}{F}-1\right)$ is a gamma distribution. Further, we acquire

$$\begin{array}{c} C=S_{\infty }\frac{{\left(\frac{B}{F}\right)}^{\frac{A}{F}-1}}{\Gamma \left(\frac{A}{F}-1\right)}, \end{array}$$

which leads to

(14) $$\begin{array}{c} H_{S}^{\infty }=S_{\infty }\frac{\xi_{S}^{\mu }}{\Gamma (\mu )}w^{-\mu -1}e^{-\frac{\xi_{S}}{w}}, \end{array}$$

where

$$\begin{array}{cc} \; & \mu =\frac{2(\zeta +\lambda +\sigma )}{\sigma }-1,\\ \; & \xi_{S}=\frac{2(m\lambda +m\zeta +\beta I_{\infty }-\gamma D)}{\sigma }, \end{array}$$

in which we require $m\lambda +m\zeta +\beta I_{\infty }-\gamma D>0$, meaning that $D<\frac{m\lambda +m\zeta +\beta I_{\infty }}{\gamma }$. The steady-state solution exhibits an inverse gamma distribution.

### 3.2. Steady-State Solution of $H_{I}\left(w,t\right)$

If we want to ask for a solution about $H_{I}\left(w,t\right)$, we need to start with Equation (6). In the same way, Equation (6) becomes

$$\begin{array}{cc} \; & \int_{R_{+}}\frac{\partial H_{I}\left(t,w\right)}{\partial t}\phi \left(w\right)dw\\ \; & =\beta I\left(t\right)\int_{R_{+}}H_{S}\left(t,w\right)\phi \left(w\right)dw-\gamma \int_{R_{+}}H_{I}\left(t,w\right)\phi \left(w\right)dw\\ \; & +\int_{R_{+}}\sum_{J\in \{S,I\}}K\left(H_{I},H_{J}\right)\left(t,w\right)\phi \left(w\right)dw, \end{array}$$

where

$$\begin{array}{cc} \; & \int_{R_{+}}\sum_{J\in \{S,I\}}K\left(H_{I},H_{J}\right)\left(t,w\right)\phi \left(w\right)dw\\ \; & =\int_{R_{+}}-m\left(t\right)\left(\zeta +\lambda \right)\frac{\partial \left(H_{I}\left(t,w\right)\right)}{\partial w}\phi \left(w\right)dw\\ \; & +\left(\zeta +\lambda \right)\int_{R_{+}}\frac{\partial \left(wH_{I}\left(t,w\right)\right)}{\partial w}\phi \left(w\right)dw\\ \; & +\int_{R_{+}}\frac{\sigma }{2}\frac{\partial^{2}}{\partial^{2}w}\left(w^{2}H_{I}\left(t,w\right)\right)\phi \left(w\right)dw. \end{array}$$

Thus, we have

$$\begin{array}{cc} \; & \int_{R_{+}}\frac{\partial H_{I}\left(t,w\right)}{\partial t}\phi \left(w\right)dw\\ \; & =\beta I\left(t\right)\int_{R_{+}}H_{S}\left(t,w\right)\phi \left(w\right)dw-\gamma \int_{R_{+}}H_{I}\left(t,w\right)\phi \left(w\right)dw\\ \; & +\int_{R_{+}}-m\left(t\right)\left(\zeta +\lambda \right)\frac{\partial \left(H_{I}\left(t,w\right)\right)}{\partial w}\phi \left(w\right)dw\\ \; & +\left(\zeta +\lambda \right)\int_{R_{+}}\frac{\partial \left(wH_{I}\left(t,w\right)\right)}{\partial w}\phi \left(w\right)dw\\ \; & +\int_{R_{+}}\frac{\sigma }{2}\frac{\partial^{2}}{\partial^{2}w}\left(w^{2}H_{I}\left(t,w\right)\right)\phi \left(w\right)dw \end{array}$$

and

$$\begin{array}{cc} \frac{\partial H_{I}\left(\omega ,t\right)}{\partial t} & =\beta I\left(t\right)H_{S}\left(t,w\right)-\gamma H_{I}\left(\omega ,t\right)\\ \; & +\frac{\partial }{\partial w}\left[\left(w(\zeta +\lambda )-m(t)(\zeta +\lambda )\right)H_{I}\left(t,w\right)\right]\\ \; & +\frac{\sigma }{2}\frac{\partial^{2}}{\partial^{2}w}\left(w^{2}H_{I}\left(t,w\right)\right). \end{array}$$

When t→∞, we assume that m(t) is a constant *m* and I(t) is a constant $I_{\infty }$, from which we obtain the equation

(15) $$\begin{array}{cc} \; & \beta I_{\infty }H_{S}^{\infty }-\gamma H_{I}^{\infty }+\left(w(\zeta +\lambda )-m(\zeta +\lambda )\right)H_{I}^{\infty }\\ \; & +\frac{\sigma }{2}\frac{\partial }{\partial w}\left(w^{2}H_{I}^{\infty }\right)=0. \end{array}$$

Bringing Equation (13) into Equation (15) yields

$$\begin{array}{cc} \; & \frac{\beta I_{\infty }}{D}H_{I}^{\infty }-\gamma H_{I}^{\infty }+\left(w(\zeta +\lambda )-m(\zeta +\lambda )\right)H_{I}^{\infty }\\ \; & +\frac{\sigma }{2}\frac{\partial }{\partial w}\left(w^{2}H_{I}^{\infty }\right)=0. \end{array}$$

We obtain

$$\begin{array}{cc} \; & \left[w\left(\zeta +\lambda +\sigma \right)-\left(\zeta +\lambda \right)m+\frac{\beta I_{\infty }}{D}-\gamma \right]H_{I}^{\infty }\\ \; & +\frac{\sigma }{2}w^{2}\frac{\partial }{\partial w}\left(H_{I}^{\infty }\right)=0. \end{array}$$

Thus, we have

$$\begin{array}{c} H_{I}^{\infty }=I_{\infty }\frac{\xi_{I}^{\mu }}{\Gamma (\mu )}w^{-\mu -1}e^{-\frac{\xi_{I}}{w}}, \end{array}$$

where

$$\begin{array}{cc} \; & \mu =\frac{2(\zeta +\lambda +\sigma )}{\sigma }-1,\\ \; & \xi_{I}=\frac{2(m\lambda +m\zeta +\gamma -\frac{\beta I_{\infty }}{D})}{\sigma }. \end{array}$$

It is worth noting that here we set the range of values for *D*. If the steady-state solution exhibits an inverse gamma distribution, we must require $m\lambda +m\zeta +\gamma -\frac{\beta I_{\infty }}{D}>0$, meaning $D>\frac{\beta I_{\infty }}{m\lambda +m\zeta +\gamma }$.

From Section 3.1, we know $D<\frac{m\lambda +m\zeta +\beta I_{\infty }}{\gamma }$. Then, we have

$$\begin{array}{cc} \; & \frac{\beta I_{\infty }}{m\lambda +m\zeta +\gamma }-\frac{m\lambda +m\zeta +\beta I_{\infty }}{\gamma }\\ \; & =\frac{\gamma \beta I_{\infty }-\left(m\lambda +m\zeta +\beta I_{\infty }\right)\left(m\lambda +m\zeta +\gamma \right)}{\gamma (m\lambda +m\zeta +\gamma )}\\ \; & =\frac{-\left[{\left(m\lambda +m\zeta \right)}^{2}+\gamma \left(m\lambda +m\zeta \right)+\beta I_{\infty }\left(m\lambda +m\zeta \right)\right]}{\gamma (m\lambda +m\zeta +\gamma )}\\ \; & <0, \end{array}$$

from which we obtain

$$\begin{array}{c} \frac{BI_{\infty }}{m\lambda +m\zeta +\gamma }<\frac{m\lambda +m\zeta +BI_{\infty }}{\gamma }. \end{array}$$

Thus, the scope of *D* satisfies

$$\begin{array}{c} \frac{\beta I_{\infty }}{m\lambda +m\zeta +\gamma }<D<\frac{m\lambda +m\zeta +\beta I_{\infty }}{\gamma }. \end{array}$$

From Equations (13) and (14), we acquire

$$\begin{array}{cc} H^{\infty } & =H_{S}^{\infty }+H_{I}^{\infty }\\ \; & =\left(1+D\right)H_{S}^{\infty }\\ \; & =S_{\infty }\left(1+D\right)\frac{\xi_{S}^{\mu }}{\Gamma (\mu )}w^{-\mu -1}e^{-\frac{\xi_{S}}{w}}, \end{array}$$

where

$$\begin{array}{cc} \; & \mu =\frac{2(\lambda +\zeta +\sigma )}{\sigma }-1,\\ \; & \xi_{S}=\frac{2(m\lambda +m\zeta +\beta I_{\infty }-\gamma D)}{\sigma }, \end{array}$$

in which $\frac{\beta I_{\infty }}{m\lambda +m\zeta +\gamma }<D<\frac{m\lambda +m\zeta +\beta I_{\infty }}{\gamma }$.

## 4. When $\frac{\beta }{\gamma }\leq 1$

Theorem 1 implies that when $\frac{\beta }{\gamma }\leq 1$, the number of the infectious I(t) at steady state (i.e., as t→∞) tends to zero. Combining with the non-negativity of the distribution function, the wealth distribution of the group approaches zero, i.e., $H_{I}^{\infty }=0$. Equation (9) becomes

$$\begin{array}{cc} \; & \frac{d}{dt}\int_{R_{+}}H_{S}\left(t,w\right)\phi \left(w\right)dw\\ \; & =\sum_{J\in \{S,I\}}\int_{R_{+}}K\left(H_{S},H_{J}\right)\left(t,w\right)\phi \left(w\right)dw, \end{array}$$

which leads to

$$\begin{array}{cc} \; & \frac{d}{dt}\int_{R_{+}}H_{S}\left(t,w\right)\phi \left(w\right)dw\\ \; & =\sum_{J\in \{S,I\}}\int_{R_{+}}K\left(H_{S},H_{J}\right)\left(t,w\right)\phi \left(w\right)dw\\ \; & =\int_{R_{+}}-m\left(t\right)\left(\zeta +\lambda \right)\frac{\partial \left(H_{S}\left(t,w\right)\right)}{\partial w}\phi \left(w\right)dw\\ \; & +\left(\zeta +\lambda \right)\int_{R_{+}}\frac{\partial \left(wH_{S}\left(t,w\right)\right)}{\partial w}\phi \left(w\right)dw\\ \; & +\int_{R_{+}}\frac{\sigma }{2}\frac{\partial^{2}}{\partial^{2}w}\left(w^{2}H_{S}\left(t,w\right)\right)\phi \left(w\right)dw. \end{array}$$

Then, we have

$$\begin{array}{cc} \frac{\partial H_{S}\left(t,w\right)}{\partial t} & =\frac{\partial }{\partial w}\left[\left(w(\zeta +\lambda )-m(t)(\zeta +\lambda )\right)H_{S}\left(t,w\right)\right]\\ \; & +\frac{\sigma }{2}\frac{\partial^{2}}{\partial^{2}w}\left(w^{2}H_{S}\left(t,w\right)\right). \end{array}$$

When t→∞, we assume that m(t) is a constant *m*. We obtain

$$\begin{array}{c} \left[w(\zeta +\lambda +\sigma )-(\zeta +\lambda )m\right]H_{S}^{\infty }+\frac{\sigma }{2}w^{2}\frac{\partial }{\partial w}\left(H_{S}^{\infty }\right)=0. \end{array}$$

When t→∞, we have $S_{\infty }+I_{\infty }=1$. Because I(t) at steady state (i.e., as t→∞) tends to zero. Thus, $S_{\infty }=1$, from which we obtain the steady-state solution

$$\begin{array}{c} H^{\infty }=H_{S}^{\infty }=\frac{\xi^{\mu }}{\Gamma (\mu )}w^{-\mu -1}e^{-\frac{\xi }{w}}, \end{array}$$

where

$$\begin{array}{cc} \; & \mu =\frac{2(\lambda +\zeta +\sigma )}{\sigma }-1,\\ \; & \xi =\frac{2(m\lambda +m\zeta )}{\sigma }. \end{array}$$

The steady-state solution exhibits an inverse gamma distribution.

## 5. Numerical Experiments

In this section, we describe the distributions of wealth at the epidemic phenomenon. We describe the temporal evolution of the susceptible and the infectious. We compare the steady-state wealth distribution and the corresponding Lorentz curves under different parameter values at the end of this section.

**Figure 1:**
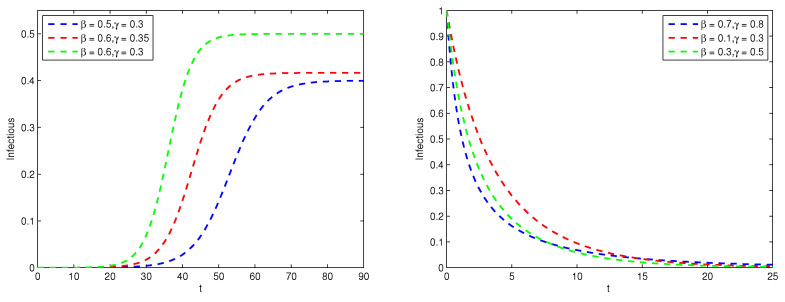
Graph of infectious proportion I(t) (The **left** is the case of $\frac{\beta }{\gamma }>1$ and the **right** is the case of $\frac{\beta }{\gamma }\leq 1$).

In Figure 1, we see that when β>γ, the number of the infectious reaches a steady-state level over time. This homeostasis exists because the recovered have no immunity and can be infected again, thus maintaining a continuous cycle of infection. When β≤γ, the SIS model behaves very differently. In this case, the disease spreads at a slower rate than it recovers. This implies that the disease does not spread rapidly enough to maintain the number of the infectious individuals, and the rate of increase in recovered individuals surpasses the rate of increase in the infectious individuals when the total population remains constant. Consequently, the number of the infectious individuals would gradually decline and eventually approach zero.

**Figure 2:**
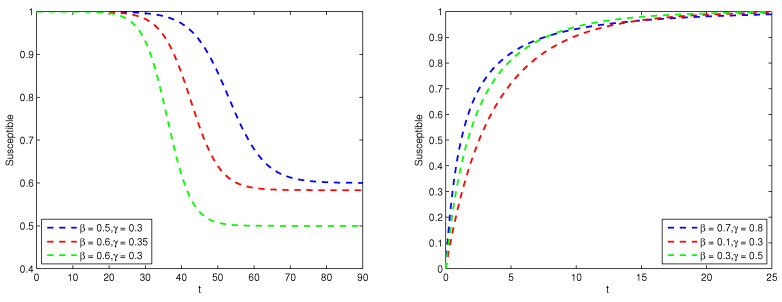
Graph of susceptible proportion S(t) (The **left** is the case of $\frac{\beta }{\gamma }>1$, and the **right** is the case of $\frac{\beta }{\gamma }\leq 1$).

Figure 2 shows the proportion of the susceptible. When β>γ, the number of the susceptible decreases to a stable value. When β≤γ, the number of the susceptible continues to increase to the total population.

**Figure 3:**
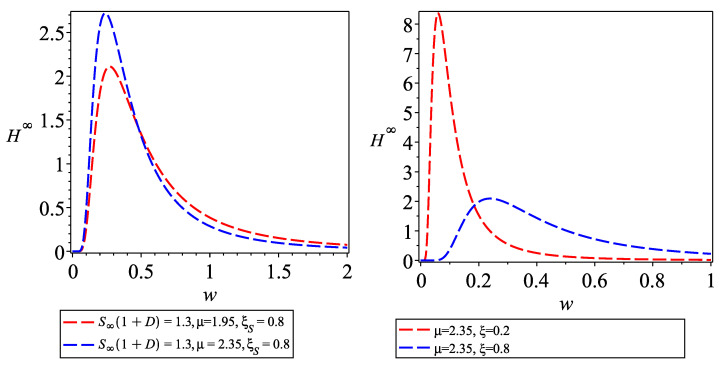
The graph of steady-state $H^{\infty }$ (The **left** is the case of $\frac{\beta }{\gamma }>1$, and the **right** is the case of $\frac{\beta }{\gamma }\leq 1$).

Figure 3 depicts the morphology of the steady-state solution with different parameters. When parameters are modified, the final steady state of the distribution function changes. Figure 3 illustrates that the steady-state wealth distribution possesses a unimodal form.

**Figure 4:**
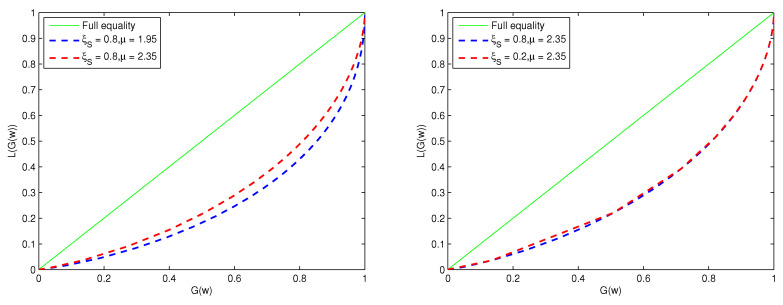
The Lorentz curve of corresponding $H^{\infty }$ (The **left** is the case of $\frac{\beta }{\gamma }>1$, and the **right** is the case of $\frac{\beta }{\gamma }\leq 1$).

Figure 4 illustrates the corresponding Lorentz curves. For the steady state $H^{\infty }$, the Lorentz curve is denoted by *L*(*G*(*w*)) [22,23]

$$L\left(G\left(w\right)\right)=\frac{\int_{0}^{w}H^{\infty }\left(z\right)zdz}{\int_{0}^{\infty }H^{\infty }\left(z\right)zdz},$$

in which

$$G\left(w\right)=\int_{0}^{w}H^{\infty }\left(z\right)dz.$$

 G(w) is a cumulative density function. Through the Lorentz curve, it is possible to visually see the situation of equality or inequality about the wealth distribution among agents. The Lorentz curve reflects the degree of inequality for the income distribution between agents. The greater the curvature of the curve, the more inequality between agents. Figure 4 illustrates that the impact of a change in μ on wealth inequality is greater than that of a change in $\zeta_{S}$. We know that the transaction coefficient λ and the proportional constant ζ are proportional to μ. When λ or ζ are larger, the μ is greater, then the wealth is more equal. This implies that a smaller wealth gap between agents leads to no extreme disparity between the rich and the poor. Therefore, if we aim to reduce the wealth gap among agents, we should consider enlarging the parameters λ or ζ.

In Figure 4, we analyze the effect of parameter ζ in expectation on wealth inequality among agents. The results of the analysis indicate that as ζ increases, wealth distribution among the agents becomes more equitable, which is not considered in [19].

## 6. Summarization

In this work, we combine the SIS model with the wealth model. Using statistical mechanics methods, we investigate the distribution of wealth in the context of epidemiological phenomena. We describe the evolution process of the wealth density function of the susceptible and the infectious. We discover that the probability density function of the infectious is related to the ratio of the interaction rate β of infections and the recovery rate γ of agents. When β≤γ, $\lim_{t\to \infty }H_{I}^{\infty }\left(w\right)=0$. When β>γ, $\lim_{t\to \infty }H_{I}^{\infty }\left(w\right)\neq 0$. Both cases are discussed. Our results show that the steady-state wealth distribution of the susceptible and the infectious patients follows a unimodal inverse gamma distribution. Finally, we analyze the ratio of two types of agents and the influence of various parameters on wealth distribution by using the numerical analysis. Investigating the impact of taxation or government control on wealth distribution in the context of infections and diseases would be our future works.

## Data Availability

No new data were created or analyzed in this study.
